# The Link Between Community-Based Violence and Intimate Partner Violence: the Effect of Crime and Male Aggression on Intimate Partner Violence Against Women

**DOI:** 10.1007/s11121-015-0567-6

**Published:** 2015-05-26

**Authors:** Ligia Kiss, Lilia Blima Schraiber, Mazeda Hossain, Charlotte Watts, Cathy Zimmerman

**Affiliations:** Department of Global Health & Development, Faculty of Public Health and Policy, London School of Hygiene & Tropical Medicine, Room 330, 15-17 Tavistock Place, London, WC1H 9SH UK; Department of Preventive Medicine, Medical School, University of São Paulo, Av. Dr. Arnaldo, 455 - Cerqueira César, CEP: 01246903 São Paulo, SP Brazil

**Keywords:** Intimate partner violence, Community violence, Epidemiology, Multilevel analysis

## Abstract

Both intimate partner violence (IPV) and community violence are prevalent globally, and each is associated with serious health consequences. However, little is known about their potential links or the possible benefits of coordinated prevention strategies. Using aggregated data on community violence from the São Paulo State Security Department (INFOCRIM) merged with WHO multi-country study on women’s health and domestic violence data, random intercept models were created to assess the effect of crime on women’s probability of experiencing IPV. The association between IPV and male aggression (measured by women’s reports of their partner’s fights with other men) was examined using logistic regression models. We found little variation in the likelihood of male IPV perpetration related to neighborhood crime level but did find an increased likelihood of IPV experiences among women whose partners were involved in male-to-male violence. Emerging evidence on violence prevention has suggested some promising avenues for primary prevention that address common risk factors for both perpetration of IPV and male interpersonal violence. Strategies such as early identification and effective treatment of emotional disorders, alcohol abuse prevention and treatment, complex community-based interventions to change gender social norms and social marketing campaigns designed to modify social and cultural norms that support violence may work to prevent simultaneously male-on-male aggression and IPV. Future evaluations of these prevention strategies should simultaneously assess the impact of interventions on IPV and male interpersonal aggression.

Violence is endemic globally, with the World Health Organization figures reporting that more than 1.6 million people are killed annually from violence and an even higher number suffer from severe violence-related health consequences (World Health Organization). Intimate partner violence (IPV) is the most common form of violence experienced by women worldwide, with an estimated global prevalence of 30 % of physical and/or sexual IPV among women over 15 years old (Devries et al. 2013; World Health Organization et al. 2013). IPV includes physical, sexual, and emotional violence, with most research to date reporting on physical and sexual violence (Devries et al. 2013). Compared to men, women report more frequent and severe levels of intimate partner violence, with acute injuries and chronic health consequences (Archer 2000; Jaden and Thoennes 2000). Conversely, compared to women, men are more likely to experience violence outside the home by unrelated individuals, who may or may not know each other (Aisenberg and Herrenkohl 2008). This violence is often defined as community violence (Krug et al. 2002) and results more often in fatal outcomes, accounting for 13.6 % of male deaths (Mercy et al. 2003).

In contrast to community violence, IPV remains largely hidden from public view (Fagan and Wexler 1987; Jackman 2002; Shileds et al. 1988). This is often attributed to the private nature and women’s reluctance to report partner violence. Episodes of IPV are often concealed from public scrutiny and are frequently perceived to be immune to community influence (Benson et al. 2004; Raghavan et al. 2006). Historically, fewer resources have been committed to preventing IPV than funds invested to address community violence. To date, there has been little robust evidence on the links between these two types of violence to inform policies or programming.

Research suggests that the perpetration of male-to-male violence and IPV victimization (male-to-female) share some similar risk factors. These include, for example, growing up in a violent home, substance abuse, social isolation, and gendered dispositions for aggressive behavior. Additionally, some personal characteristics such as poor behavioral control and low sense of self-worth appear to be associated with perpetration of both types of violence (Abramsky et al. 2011; Anderson and Bushman 2002; Fagan and Wexler 1987; Jewkes 2002; Krug et al. 2002).

Research has also indicated that there are different types of perpetrators, with a diverse range of motivations for violent behaviors. Over the past two decades, a number of studies have focused on examining patterns of violence perpetration by an intimate partner and exploring the diversity of targets of interpersonal violence (e.g., family only, others outside the family) (Anderson and Anderson 2008; Cavanaugh and Gelles 2005; Holtzworth-Munroe and Meehan 2004; Thijssen and Ruiter 2011). This research has suggested, for instance, that the subtypes of male IPV perpetrators vary by three general factors: (1) severity and frequency of violence, (2) generality of targets of violence (family only or outside the family), and (3) personality disorder and mental health issues. Researchers have found that men who are violent towards their female partners are not necessarily aggressive outside the home and may target female partners exclusively (Anderson and Bushman 2002; Holtzworth-Munroe and Meehan 2004). Hostility to women and the exclusivity of female targets seem to be common only among a certain subtype of male perpetrators who would also be more likely to adhere to traditional notions of gender roles, have abusive attitudes towards women, have experienced violence in their childhood, associate with deviant peers, have more substance abuse problems, and engage in generally antisocial behavior (Anderson and Anderson 2008; Holtzworth-Munroe and Meehan 2004). Perpetration of intimate partner violence also appears to be influenced by social gender norms that reinforce traditional notions of manhood, including a focus on success and power, multiple sexual partners, homophobia, and use of controlling tactics within relationships (Kiss et al. 2012; Mankowski and Maton 2010; Peralta et al. 2010).

Aggressive individuals are more likely to engage in generally antisocial behavior (Thijssen and Ruiter 2011) and to interact with violent peers creating situations where the use of violence becomes a normative behavior (Silverman and Williamson 1997). In this sense, local social norms that condone violence may also have a powerful influence on violent behavior (Bogat et al. 2005; Sampson and Lauritsen 1990). Many theories have called attention to a “culture of violence” effect, which suggests that people living in a violent context would share a cognitive landscape embedded in violence and be more prone to aggressive behavior. Similarly, research also suggests the presence of an endogenous social effect on IPV in which living in a neighborhood with high IPV rates increases a woman’s likelihood of experiencing this type of violence, even after adjusting for other individual-level factors (McQuestion 2002). However, the association of non-lethal IPV with neighborhood structural characteristics appears to be relatively weak (Browning 2002).

Using the WHO multi-country study on women’s health and domestic violence data on IPV and male intimate partners’ behavior in São Paulo and crime data from the Security Secretary for the State of São Paulo (SSP/SP), we examined the relationship between community violence and IPV. We aimed to assess the association between levels of community violence and women’s risk of experiencing intimate partner violence. Specifically, we sought to answer the following questions: Does living in a violent neighborhood increase a woman’s probability of IPV? Does having a male partner who is aggressive towards other men increase a woman’s likelihood of experiencing IPV?

## Methods

### Study Setting and Participants

São Paulo is a city of more than 11 million inhabitants located in the southeast region of Brazil (Instituto Brasileiro de Geografia e Estatistica (Brazilian Institute of Geography and Statistics) 2010). The World Health Organization (WHO) multi-country population survey of women found that 9.3 % of women in São Paulo who had been in an intimate relationship had experienced physical and/or sexual violence in the last year (Garcia-Moreno et al. 2006). This rate was lower than several of the less developed countries in the WHO multi-country study, but higher than that documented in Japan (3.8 %) or in New Zealand (5.6 %) (Fanslow and Robinson 2004). In contrast, the figures for homicides in Brazil were much higher than those in most industrialized countries (Moser 2005). In 2000, homicide rates per 100,000 inhabitants in Germany and France were 1.2 and 1.8, respectively, and 5.5 in the USA (2005), while in Brazil, the rate was 25.7 (Waiselfisz 2007). In 1999, the homicide rate for the city of São Paulo was 35.3 homicides per 100,000 inhabitants. This pattern makes São Paulo a unique case study to explore the effect of crime and male aggression on intimate partner violence.

We used the Brazilian data from the 2000 WHO multi-country study on women’s health and domestic violence which was conducted in partnership with the Medical School, University of São Paulo. We selected a representative sample of women living in the city of São Paulo (Garcia-Moreno et al. 2005; Schraiber et al. 2007) by multi-stage sampling by clusters (Silva et al. 2003). Sample units were selected in three stages using probability proportional to size. In the first stage, 72 census tracts were randomly selected from the probability matrix of 263 tracts which were ranked by census blocks and level of education of household heads. In the second stage, 30 households were selected in each tract. Finally, in each household with female residents aged 15 to 49, one woman was randomly selected to be interviewed. The household response rate was 94.4 %, and individual response rate was 89.9 %. The sample was weighted to account for the lack of equiprobability in sampling since only one woman was selected per household with different numbers of female inhabitants. Data on rates of violent crime were derived from the SSP/SP for the same 72 tracts and merged into the WHO dataset.

Census tracts are the smallest geographical unit defined by the Brazilian Institute of Geography and Statistics (IBGE). The boundaries between tracts are drawn to include 250 households. Although the boundaries between neighborhoods are not defined by socioeconomic characteristics, the inclusion of a small number of neighboring households tends to guarantee a certain degree of social homogeneity. Hereafter, census tracts will be referred to as neighborhoods.

Data on homicides and on property crimes were obtained from the SSP/SP. These data are collected by the System of Information for Criminal Occurrences, which is unified data and intelligence system resulting in a database system of crime geo-referencing (Tella et al. 2009).

Bayesian crime rates were calculated for 71 neighborhoods sampled in the WHO study. One cluster was excluded from the analysis because of unavailability of crime data. The Bayesian empirical rate is a weighted mean between the crude local rate and the global regional rate. If the locality has a considerable sized population, the rate has small variability. If the locality has a small population, the crude rate estimation has greater variance and less weight is attributed to this unstable rate, rendering the value of the Bayesian rate closer to the expected value of a randomly selected area (Marshall 1996).

### Ethics

Ethical approval for the study was granted by the Ethics Committee in Research of the Medical School University of São Paulo, Brazil, and by the Brazilian National Commission in Research Ethics. Further analysis of the dataset under a UK Economic and Social Sciences Research Council (ESRC) project was approved by the Ethics Committee of the London School of Hygiene and Tropical Medicine (no. 5670) in February 2010.

### Measures

#### Intimate Partner Violence

IPV was measured using the WHO violence against woman questionnaire (Garcia-Moreno et al. 2005, 2006). The instrument included five items on specific acts of physical violence (slapped or threw something that could hurt; pushed or shoved; hit with fist or something that could hurt; kicked, dragged, or beat up; chocked or burnt; threatened to use or used a weapon) and three items on sexual violence (physically forced to have sex when did not want to; had sex because was afraid of what partner might do; forced to do something sexual that she found degrading or humiliating). It was validated in Brazil and deemed adequate for estimating intimate partner violence against women (Schraiber et al. 2010). The timing of physical and sexual violence was assessed using an exposure table dating the first and last episodes of violence by each partner. The IPV variable was coded as positive if there was an overlap between episodes of violence and the time since the woman started living in the neighborhood where she was interviewed. Emotional violence (insulted or made her feel bad about herself; belittled or humiliated her; scared or intimidated her on purpose; threatened to hurt) was not included in the IPV variable construction because the timing of episodes was not available for this type of abuse.

Three indicators were used to assess community violence: level of crime in the neighborhood (or criminal violence), crime victimization in the neighborhood, and male-to-male violence (male partner fights with other men).

#### Level of Crime in the Neighborhood (Based on Brazilian Official Crime Statistics for the State of São Paulo (INFOCRIM))

Data on violent crime were used to characterize levels of community violence in each neighborhood. Four variables on homicides were included, corresponding to the Brazilian legal classification for criminal acts of homicide: (1) intentional homicide, (2) unintentional homicide, (3) qualified homicide, and (4) attempted homicide. Property crime was measured using the following variables: (5) robbery with a lethal outcome, (6) stealing, (7) theft, and (8) qualified theft. Box 1 defines each crime according to the Brazilian Penal Code (Governo Federal Presidencia da Republica, decreto-lei no. 2.848, 7 December 1940).

Box 1. Crime variables and definitions according to the Brazilian Penal Code (Governo Federal Presidencia da Republica, decreto-lei no. 2.848, 7 December 1940)

**Table:** 

Intentional homicide	An act committed with the intention to kill the victim or when death occurs as its predictable consequence
Unintentional homicides	An action which did not have murder as its final purpose or as a predictable consequence
Qualified homicide	One or more of the following circumstances apply: (a) the crime is committed for payment or compensation; (b) the crime involves malicious intent; (c) the intent is considered futile or a disproportionate reaction to a relatively minor situation; (d) the offender behaves with cruelty which results in serious harm to the public; (e) the homicide involves cheating, misrepresentation, or any means that make it difficult or impossible for the victim to defend themselves; or (f) the crime aims to ensure the execution, hiding, impunity, or advantages of another crime
Attempted homicide	An unsuccessful attempt to kill someone
Robbery with a fatal outcome	A situation when violence is used to conduct the robbery and results in the death of the victim
Qualified theft	Defined by (1) the destruction of obstacles to the crime; (2) the abuse of trust, employment of fraud, dexterity, or skills; (3) the use of fake keys; and (4) more than one participating individual
Stealing	The act of intentionally taking the personal property of another without consent through the use of severe threat or violence

Composite measures for homicide and crimes against property were created as the sum of the indicators for each type of crime on the scale of 1:100,000. The four homicide indicators (intentional, unintentional, qualified, and attempted) were combined as one single homicide variable. The indicators for property crime were calculated as an aggregate of the indicators on robbery with a lethal outcome, qualified theft, and stealing. To examine the association between IPV and neighborhood crime, variables divided into quintiles were created for intentional homicides and for property crimes within each of the study neighborhoods.

#### Crime Victimization in the Neighborhood

The WHO survey data on crime victimization was included in the analysis. A categorical variable was created by summing up the number of households with reports of crime victimization within each neighborhood in the 4 weeks prior to the interview. The categories included the following: 0 crime reported, 1 crime, and 2+ crimes for each neighborhood.

#### Male-to-Male Violence (Male Partner Fights with Other Men)

Women were asked whether their male intimate partner was ever involved in physical fights with other men since she had known him. A small number of women reported that they did not know (*n* = 5/923). These cases were considered missing and excluded from the analysis.

#### Confounders

Potential confounders measuring partner’s characteristics and behaviors were identified based on a previous work with the WHO dataset (Kiss et al. 2012) and using directed acyclic graphs. These confounders included heavy alcohol use, partner education, and women’s time living in the neighborhood. Heavy alcohol use was classed positive for partners who were reported drunk at least once a month. Partner’s education was measured as numbers of completed years of schooling. A separate variable was created for women who did not know their partner’s number of years in formal education.

Women’s number of years living in the neighborhood was included in the models to control for increased exposure to IPV, since we measured only episodes that happened while the woman was living in the community.

### Data Analysis

Descriptive statistics of individual- and contextual-level exposure variables were calculated and presented alongside logit estimates of individual-level odds ratios adjusted by the number of years living in the neighborhood. *p* values for the association between male partner variables were calculated using chi-square test.

Random intercept models with IPV as the outcome variable and crime as predictors were used to examine if a variation in neighborhood-level crime rates was associated with variations in the likelihood of individual IPV experiences. If the higher-level variation was significant, the analysis examined if this effect persisted when individual-level variables were included in the model, thus leading to the conclusion that contextual effects matter. If the association was not significant, we could conclude that living in a violent neighborhood did not significantly affect a woman’s risk of IPV in this sample. We calculated the median odds ratio (MOR) to quantify the variation between clusters as a function of the cluster variance to facilitate parameter interpretation (Larsen and Merlo 2004). We also calculated the *p* values for the likelihood ratio test for the between-neighborhood variance and the intra-class correlation coefficient (ICC). The number of integration points for the random intercept models was tested, and the default of 7 was deemed adequate.

**Fig. 1:**
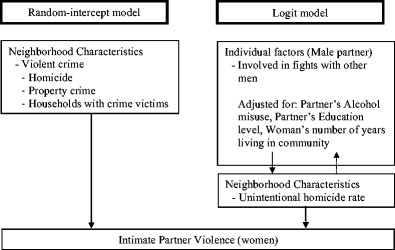
Analytical strategy for the study

Logistic regression was used to examine the effect of a partner’s involvement in fights with other men on women’s reports of IPV experiences. The model was adjusted for partner’s education and alcohol misuse—the potential confounders identified based on previous work by our team (Kiss et al. 2012) and through the use of directed acyclic graphs (Fleisher and Diez-Roux 2008; Shrier and Platt 2008). The Bayesian rate for each community violence variable was then included in the model to verify whether a high-level variation in crime would affect the strength of the association between the partner’s individual-level variables and IPV. If the strength of the association was affected by the inclusion of community-level crime variables, we could establish that living in a violent neighborhood influences the likelihood of IPV beyond individual-level factors in this sample. The analysis was performed with Stata 13. Figure 1 describes the analytical stragey for the study.

## Results

The mean age of women in the study sample was 33.6 years (SD = 8.8). Almost half of the women had only primary or elementary education (48.0 %), having completed the first 8 years of compulsory basic schooling. The majority of women were in an intimate relationship, with 52.1 % currently married, 20.3 % co-habiting with a man, and 16.4 % living apart. The mean age of intimate partners was 36.6 years (SD = 9.9), and 44.8 % of the male partners had only primary education.

In 2000, São Paulo had an average intentional homicide rate of 46 per 100,000. More than one in six women (17.3 %) experienced IPV while living in the neighborhood where they were interviewed. The lifetime prevalence of physical and/or sexual IPV in São Paulo was 28.9 %, with 27.2 % of women reporting physical violence, 10.1 % sexual violence, and 41.8 % emotional violence (Table 1).

**Table 1:** Frequency of women exposed to individual- and neighborhood-level indicators of community violence according to levels of intimate partner violence (IPV), adjusted odds ratio (AOR) (95 % CI), and *p* values for associations with IPV against women (*n* = 923)

	*N* (%)	Frequency of IPV (past year) *N* (%)		AOR^a^ (95 % CI)	*p* value
Variable		Yes	No		
Individual level					
Partner in fights with men					
Yes	100 (10.7)	64 (64.0)	36 (36.0)	3.24 (2.03–5.17)	0.000
Neighborhood level					
Neighborhood homicide rate quintiles (rate per 100,000 inhabitants)					
1st quintile (5 to 27 homicides)	191 (20.7)	38 (19.9)	153 (80.1)	1	
2nd quintile (29 to 45 homicides)	198 (21.5)	46 (23.2)	152 (76.8)	1.15 (0.68–1.94)	0.463
3rd quintile (51 to 70 homicides)	175 (19.0)	21 (12.0)	154 (88.0)	0.52 (0.28–0.96)	0.055
4th quintile (72 to 101 homicides)	179 (19.4)	29 (16.2)	150 (83.8)	0.76 (0.43–1.35)	0.378
5th quintile (103 to 154 homicides)	180 (19.5)	28 (15.6)	152 (84.4)	0.72 (0.41–1.28)	0.298
Neighborhood property crime rate quintiles (rate per 100,000 inhabitants)					
1st quintile (12 to 69 crimes)	202 (21.9)	33 (16.3)	169 (83.7)	1	
2nd quintile (71 to 102 crimes)	182 (19.7)	31 (17.0)	151 (83.0)	1.05 (0.60–1.84)	0.853
3rd quintile (103 to 122 crimes)	181 (19.6)	31 (17.1)	150 (82.9)	1.05 (0.60–1.84)	0.861
4th quintile (124 to 147 crimes)	199 (21.6)	49 (24.6)	150 (75.4)	1.67 (1.00–2.80)	0.049
5th quintile (148 to 195 crimes)	159 (17.2)	18 (11.3)	141 (88.7)	0.66 (0.35–1.24)	0.197
Households with crime victims in the previous 4 weeks within the neighborhood					
No of households with crime reported	372 (39.6)	71 (19.1)	301 (80.9)	1	
One household with crime reported	391 (41.6)	66 (16.9)	325 (83.1)	0.86 (0.57–1.32)	0.502
Two or more households with crime reported	177 (18.8)	26 (14.7)	151 (85.3)	0.72 (0.42–1.26)	0.250

^a^Adjusted for the number of years living in the community

Neighborhoods in the lower homicide rate quintile presented a Bayesian estimate of between 5 and 27 homicides per 100,000, while the homicide rate estimated for higher quintiles varied from 103 to 154 homicides per 100,000. Property crimes had higher rates within neighborhoods, with those in the 1st quintile having a crime rate of 12 to 69 per 100,000 inhabitants and those in the 5th having between 148 to 195 crimes. Three in five neighborhoods (60.4 %) had at least one household that reported a crime victim in the previous 4 weeks. None of the contextual-level crime variables were significantly associated with IPV against women at the individual level. However, having a male partner who was involved in fights with other men was significantly associated with women’s reports of IPV.

The median OR findings revealed some interesting patterns. For example, if a woman moved to an area with a higher probability of unintentional homicides, her likelihood of experiencing IPV increased 1.26 times. If she moved to a neighborhood with a higher probability of property crimes, her likelihood of experiencing IPV increased by 1.18. In a neighborhood with high levels of victimization (households with a crime reported in the last 4 weeks), the probability of IPV increased around 1.41 times (Table 2). However, the between-neighborhood variance was not significant for any type of crime. The association between crimes and IPV also showed a low variation at the neighborhood level, with ICCs of between 0.02 and 0.03. Additionally, the variability at the contextual level was smaller in the models including the crime variables than that in the baseline model for IPV. We can, therefore, conclude that living in a violent neighborhood did not significantly affect a woman’s risk of IPV in São Paulo in 2000.

**Table 2:** Frequency of violent crimes and results from random intercept model and measures of variation at the neighborhood level for the association between violent crime and IPV against a woman (*N* = 923 individuals within 71 clusters)

Variables	Baseline model^a^	IPV homicide model^b^	IPV crimes against property model^b^	IPV households with crime victim model^b^
Median odds ratio (MOR)	1.45	1.26	1.18	1.41
Intra-class correlation coefficient (ICC)	0.04	0.02	0.01	0.03
Likelihood test for between-neighborhood variance (*p* value)	0.06	1.26	0.37	0.08

^a^Null model, without exposure variables

^b^Models adjusted for the number of years living in the community

Women living in areas with higher urban violence indicators also showed higher rates of having male partners who were involved in physical fights with other men. Across the quintiles of homicides, there was a slight increase in the proportion of partners who were ever involved in physical fights with other men, ranging from 9.6 % in the first quintile to 12.2 % in the fifth one. However, this difference was not statistically significant (*p* = 0.92).

Among the women with partners ever involved in fights with other men, 38 % reported that partners were drunk at least once a month (*p* = 0.00). Having partners involved in fights with other men was not significantly associated with their level of education (*p* = 0.15). Partners with little or no education were reported to be drunk more often (22.5 %) when compared with men who completed primary (14 %) and secondary education (6 %) (*p* = 0.00) (Table 3).

**Table 3:** Partner involvement in fights with other men and woman’s likelihood of IPV (frequency, crude OR, and adjusted OR for models 1 and 2) (*n* = 923)

Variable	Frequency (%)	Crude OR (95 % CI)	Model 1 (individual-level variables only) Adjusted OR (95 % CI)^a^	Model 2 (including neighborhood unintentional homicide rate) Adjusted OR (95 % CI)^a^
Partner involved in fights with other men^b^	10.7	3.20 (2.04–5.01)	2.68 (1.58–4.53)	2.58 (1.52–4.39)

^a^Adjusted for partner alcohol use, partner education, and number of years living in the community

^b^Five are missing

Women who reported that their current or most recent partner had been in fights with other men had 3.2 greater odds of experiencing IPV compared to those whose partners were never in fights (crude odds ratio (OR) = 3.20; 95 % confidence interval (CI) = 2.04–5.01). After adjustment for other partner’s behaviors and experiences, this odds ratio decreased by 19 % (adjusted OR = 2.68; 95 % CI = 1.58–4.53) but remained highly significant. In the final model including neighborhood unintentional homicide rate, women whose partners were involved in fights with other men had 2.58 greater odds of experiencing IPV. The inclusion of neighborhood-level violence factors did not significantly change this association (adjusted OR = 2.58; 95 % CI = 1.52–4.39).

## Discussion

Despite high levels of variation in the rates of community violence in this sample, IPV was not significantly associated with neighborhood levels of violent crime in São Paulo. As such, our findings do not support the hypothesis of the impact of a violent social context on women’s risk of experiencing IPV in São Paulo. Rather, individual-level partner variables seemed to be more significant determinants in the probability that a woman will experience IPV by her male partner, even after accounting for contextual indicators of crime and violence in her neighborhood.

These results suggest that male interpersonal aggression (physical fights) and violent crime may differ in their relation to IPV perpetration against women. Prevention interventions for these various types of violence would benefit from further evidence to understand the motivations for different types of violent behaviors, especially considering the range of targets and the criminal or impulsive nature of violent acts (Anderson and Bushman 2002).

Emerging evidence on violence prevention has suggested some promising avenues for primary prevention that address common risk factors for both perpetration of IPV and male interpersonal violence. These factors include growing up in a violent home, harmful use of alcohol, gendered motivations to aggressive behavior, and social norms condoning violence (Abramsky et al. 2011; Anderson and Bushman 2002; Fagan and Wexler 1987; Jewkes 2002; Krug et al. 2002). Specifically, strategies that may work to prevent both male-on-male aggression and IPV may include interventions such as parent training and parent-child programs, early identification and effective treatment of conduct and emotional disorders, alcohol abuse prevention and treatment, community-based multilevel interventions to shift gendered social norms, and social marketing campaigns designed to modify social and cultural norms that support violence (Abramsky et al. 2014; Hossain et al. 2014; World Health Organization and Liverpool JMU Centre for Public Health 2010; World Health Organization and London School of Hygiene and Tropical Medicine 2010). Future evaluations of these prevention strategies should simultaneously assess the impact of the interventions on IPV perpetration and male-on-male aggression.

There are several limitations to this study. Criminal statistics have some intrinsic reporting bias, as victims and witnesses do not always report crimes and the police may also fail to record all crimes. Sometimes, an increase in crimes recorded by the police in a given area can reflect better policing in this area rather than a rise in violent crimes. This may hold true for thefts, robberies, and attempted homicides, where victims may choose not to report the crime or the evidence can be more easily hidden or forgotten. However, when considering crimes with a fatal outcome, the reliability tends to increase (Sampson et al. 1977). Nonetheless, official criminal statistics offer the advantage of large representative samples disaggregated by small geographical units, and the possibility of future comparative longitudinal analysis. Additionally, using secondary data as indicators of community violence avoids multicollinearity (where two or more predictor variables are highly correlated), which could pose a problem for contextual-level variables created from the surveys’ individual-level data. For this reason (multicollinearity), the variable on crime victimization in the neighborhoods should be interpreted with caution. This variable was included because it is a reliable indicator of level of victimization in the neighborhoods, including crimes that may not have been reported to the police.

The variables on partners’ behaviors are reliant upon women’s reports and may be affected by information and recall bias. Women may not know about their partner’s past behavior and may not report violence because of embarrassment, fear, or self-blame. For ethical reasons, the WHO survey did not interview both intimate partners (Watts et al. 1999).

More broadly, the lack of longitudinal data on IPV limits our ability to draw conclusions on the extent to which IPV is susceptible to changes in the social environment. However, if gender power imbalances are indeed a major determinant of IPV, as research indicates (Jewkes 2002; Michalski 2004; Rutherford et al. 2007), rates should fluctuate as a result of changes in the sociopolitical and cultural context related to gender. These contextual shifts may take more time, even generations to be incorporated into individual behavior versus potentially more rapid or wider-reaching impact on the triggers for criminal behaviors (e.g., reductions of the presence of drug trafficking, firearms, and imprisonment rates). Research on the social influences and historical trends on IPV will benefit from cohort data and the disaggregation of crime information by sex.

Finally, even if the association between IPV and community violence was not significant for São Paulo in the year 2000, this may not be the case for other sites. The hypothesis tested in this study may hold true in different settings. The field would benefit from learning more about social norms that may be in the causal pathway for different types of violence.

Despite these limitations, to our knowledge, our study is the first to use multilevel analysis to explore contextual effects of crime on IPV, providing valuable insights to policy development in the field of violence prevention. Findings suggest that individual-level partner’s behavior may be a strong predictor of IPV. Further studies should explore the nature of these associations at different levels across time. Meanwhile, policy-making and programming on interpersonal violence can offer more immediate responses in tackling male violent behavior by combining strategies to prevent male aggression in the streets and in the home.

## References

[CR1] Abramsky T, Watts CH, Garcia-Moreno C, Devries K, Kiss L, Ellsberg M (2011). What factors are associated with recent intimate partner violence? Findings from the WHO multi-country study on women’s health and domestic violence. BMC Public Health.

[CR2] Abramsky T, Devries K, Kiss L, Nakuti J, Kyegombe N, Starmann E (2014). Findings from the SASA! Study: A cluster randomized controlled trial to assess the impact of a community mobilization intervention to prevent violence against women and reduce HIV risk in Kampala, Uganda. BMC Medicine.

[CR3] Aisenberg E, Herrenkohl T (2008). Community violence in context risk and resilience in children and families. Journal of Interpersonal Violence.

[CR4] Anderson C, Anderson K (2008). Men who target women: Specificity of target, generality of aggressive behavior. Aggressive Behavior.

[CR5] Anderson CA, Bushman BJ (2002). Human aggression. Annual Review of Psychology.

[CR6] Archer J (2000). Sex differences in aggression between heterosexual partners: A meta-analytic review. Psychological Bulletin.

[CR7] Benson M, Fox G, Maris AD, Wyk JV (2004). Neighbourhood disadvantage, individual economic distress and violence against women in intimate relationships. Journal of Quantitative Criminology.

[CR8] Bogat A, Leahy K, Eye AV, Maxwell C, Levendosky A, Davidson W (2005). The influence of community violence on the functioning of women experiencing domestic violence. American Journal of Community Psychology.

[CR9] Brazilian Institute of Geography and Statistics (Instituto Brasileiro de Geografia e Estatistica). (2010). IBGE cities. http://www.cidades.ibge.gov.br/xtras/perfil.php?lang=_EN&codmun=355030&search=sao-paulo|sao-paulo. Accessed 16 July 2012.

[CR10] Brazilian Penal Code (Código Penal - CP) - DL-002.848-1940. *Law number 2848* (decreto-lei no. 2848), 7 December 1940.

[CR11] Browning C (2002). The span of collective efficacy: Extending social disorganization theory to partner violence. Journal of Marriage and Family.

[CR12] Cavanaugh MM, Gelles RJ (2005). The utility of male domestic violence offender typologies new directions for research, policy, and practice. Journal of Interpersonal Violence.

[CR13] Devries K, Mak J, García-Moreno C, Petzold M, Child J, Falder G (2013). The global prevalence of intimate partner violence against women. Science.

[CR14] Fagan J, Wexler S (1987). Crime at home and in the streets: The relationship between family and stranger violence. Violence and Victims.

[CR15] Fanslow J, Robinson E (2004). Violence against women in New Zealand: Prevalence and health consequences. New Zealand Medical Journal.

[CR16] Fleisher N, Diez-Roux A (2008). Using directed acyclic graphs to guide analyses of neighbourhood health effects: An introduction. Journal of Epidemiology and Community Health.

[CR17] Garcia-Moreno, C., Jansen, H. A., Ellsberg, M., Heise, L., & Watts, C. H. (2005). *WHO multi-country study on women’s health and domestic violence against women: Initial results on prevalence, health outcome and women’s response*. In: World Health Organization (Ed.). Geneva.

[CR18] Garcia-Moreno C, Jansen HA, Ellsberg M, Heise L, Watts CH (2006). Prevalence of intimate partner violence: Findings from the WHO multi-country study on women’s health and domestic violence. The Lancet.

[CR19] Holtzworth-Munroe A, Meehan JC (2004). Typologies of men who are maritally violent: Scientific and clinical implications. Journal of Interpersonal Violence.

[CR20] Hossain M, Zimmerman C, Kiss L, Abramsky T, Kone D, Bakayoko-Topolska M (2014). Working with men to prevent intimate partner violence in a conflict-affected setting: A pilot cluster randomized controlled trial in rural Cote d’Ivore. BMC Public Health.

[CR21] Jackman MR (2002). Violence in social life. Annual Review of Sociology.

[CR22] Jaden P, Thoennes N (2000). Prevalence and consequences of male-to-female and female-to-male intimate partner violence as measured by the national violence against women survey. Violence Against Women.

[CR23] Jewkes R (2002). Intimate partner violence: Causes and prevention. The Lancet.

[CR24] Kiss L, Schraiber L, Heise L, Zimmerman C, Gouveia N, Watts C (2012). Gender based violence and socioeconomic inequalities: Does living in more deprived neighbourhoods increase women’s risk of intimate partner violence?. Social Sciences & Medicine.

[CR25] Krug, E., Dahlberg, L., Mercy, J., & Zwi, A. (2002). *World report on violence and health.* In: *World Health Organization* (Ed.). Geneva.

[CR26] Larsen K, Merlo J (2004). Appropriate assessment of neighbourhood effects on individual health: Integrating random and fixed effects in multilevel logistic regression. American Journal of Epidemiology.

[CR27] Mankowski E, Maton K (2010). A community psychology of men and masculinity: Historical and conceptual review. American Journal of Community Psychology.

[CR28] Marshall R (1996). Mapping disease and mortality rates using empirical Bayes estimators. Journal of Royal Statistical Society Series C Applied Statistics.

[CR29] McQuestion M (2002). Endogenous social effects on intimate partner violence in Colombia. Social Science Research.

[CR30] Mercy JA, Krug EG, Dahlberg LL, Zwi AB (2003). Violence and health: The United States in a global perspective. American Journal of Public Health.

[CR31] Michalski JH (2004). Making sociological sense out of trends in intimate partner violence: The social structure of violence against women. Violence Against Women.

[CR32] Moser CON (2005). Latin American urban violence as a development concern: Towards a framework for violence reduction. World Development.

[CR33] Peralta R, Tuttle L, Steele J (2010). At the intersection of interpersonal violence, masculinity, and alcohol use: The experiences of heterosexual male perpetrators of intimate partner violence. Violence Against Women.

[CR34] Raghavan C, Mennerich A, Sexton E, James SE (2006). Community violence and its direct, indirect and mediating effects on intimate partner violence. Violence Against Women.

[CR35] Rutherford A, Ziwi AB, Grove NJ, Buchart A (2007). Violence: A priority for public health?. Journal of Epidemiology and Community Health.

[CR36] Sampson R, Lauritsen J (1990). Deviant lifestyles, proximity to crime, and the offender-victim link in personal violence. Journal of Research in Crime and Delinquency.

[CR37] Sampson RJ, Raudenbush SW, Earls F (1977). Neighborhoods and violent crime: A multilevel study of collective efficacy. Science.

[CR38] Schraiber LB, D’OliveiraI AFPL, França-Junior I, DinizI S, Portella AP, Ludermir AB (2007). Prevalence of intimate partner violence against women in regions of Brazil. Revista de Saúde Pública.

[CR39] Schraiber LB, Latorre RM, Franca I, Segri N, D’Oliveira AF (2010). Validity of the WHO VAW study instrument for estimating gender-based violence against women. Revista de Saúde Pública.

[CR40] Shileds NM, McCall GJ, Hanneke CR (1988). Patterns of family and nonfamily violence: Violent husbands and violent men. Violence and Victims.

[CR41] Shrier I, Platt RW (2008). Reducing bias through directed acyclic graphs. BMC Medical Research Methodology.

[CR42] Silva N, Cinga T, Quintanilha J (2003). Master sample and geoprocessing: Technologies for household surveys. Revista de Saúde Pública.

[CR43] Silverman J, Williamson G (1997). Social ecology and entitlements involved in battering by heterosexual college males: Contributions of family and peers. Violence Victims.

[CR44] Tella RD, Edwards S, Schargrodsky E (2009). The economics of crime: Lessons for and from Latin America.

[CR45] Thijssen J, Ruiter CD (2011). Identifying subtypes of spousal assaulters using the B-SAFER. Journal of Interpersonal Violence.

[CR46] Waiselfisz J (2007). Map of violent deaths. Estudo Avançados.

[CR47] Watts, C., Ellsberg, M., & García-Moreno, C. (1999). *Putting women’s safety first: Ethical and safety recommendations for research on domestic violence against women*. In: World Health Organization. (Ed.). Geneva.

[CR48] World Health Organization, & Liverpool JMU Centre for Public Health. (2010). *Violence prevention: The evidence*. In: World Health Organization (Ed.). Geneva.

[CR49] World Health Organization, & London School of Hygiene and Tropical Medicine. (2010). *Preventing intimate partner and sexual violence against women: Taking action and generating evidence*. In: World Health Organization (Ed.). Geneva.

[CR50] World Health Organization, London School of Hygiene and Tropical Medicine, & South African Medical Research Council. (2013). *Global and regional estimates of violence against women: Prevalence and health effects of intimate partner violence and non-partner violence*. In: World Health Organization (Ed.). Geneva.

